# *Stenotrophomonas maltophilia* Infections in a General Hospital: Patient Characteristics, Antimicrobial Susceptibility, and Treatment Outcome

**DOI:** 10.1371/journal.pone.0037375

**Published:** 2012-05-18

**Authors:** George Samonis, Drosos E. Karageorgopoulos, Sofia Maraki, Panagiotis Levis, Dimitra Dimopoulou, Nikolaos A. Spernovasilis, Diamantis P. Kofteridis, Matthew E. Falagas

**Affiliations:** 1 Department of Internal Medicine, University Hospital of Heraklion, Heraklion, Crete, Greece; 2 Alfa Institute of Biomedical Sciences (AIBS), Marousi, Athens, Greece; 3 Hellenic Center for Disease Control and Prevention, Marousi, Athens, Greece; 4 Department of Clinical Microbiology, University Hospital of Heraklion, Heraklion, Crete, Greece; 5 Department of Medicine, Henry Dunant Hospital, Athens, Greece; 6 Department of Medicine, Tufts University School of Medicine, Boston, Massachusetts, United States of America; Los Angeles Biomedical Research Institute, United States of America

## Abstract

**Introduction:**

*Stenotrophomonas maltophilia* is acquiring increasing importance as a nosocomial pathogen.

**Methods:**

We retrospectively studied the characteristics and outcome of patients with any type of *S. maltophilia* infection at the University Hospital of Heraklion, Crete, Greece, between 1/2005–12/2010. *S. maltophilia* antimicrobial susceptibility was tested with the agar dilution method. Prognostic factors for all-cause in-hospital mortality were assessed with multivariate logistic regression.

**Results:**

Sixty-eight patients (median age: 70.5 years; 64.7% males) with *S. maltophilia* infection, not related to cystic fibrosis, were included. The 68 patients were hospitalized in medical (29.4%), surgical (26.5%), hematology/oncology departments (23.5%), or the intensive care units (ICU; 20.6%). The most frequent infection types were respiratory tract (54.4%), bloodstream (16.2%), skin/soft tissue (10.3%), and intra-abdominal (8.8%) infection. The *S. maltophilia-*associated infection was polymicrobial in 33.8% of the cases. *In vitro* susceptibility was higher to colistin (91.2%), trimethoprim/sulfamethoxazole and netilmicin (85.3% each), and ciprofloxacin (82.4%). The empirical and the targeted treatment regimens were microbiologically appropriate for 47.3% and 63.6% of the 55 patients with data available, respectively. Most patients received targeted therapy with a combination of agents other than trimethoprim/sulfamethoxazole. The crude mortality and the mortality and the *S. maltophilia* infection-related mortality were 14.7% and 4.4%, respectively. ICU hospitalization was the only independent prognostic factor for mortality.

**Conclusion:**

*S. maltophilia* infection in a general hospital can be associated with a good prognosis, except for the patients hospitalized in the ICU. Combination reigmens with fluoroquinolones, colistin, or tigecycline could be alternative treatment options to trimethoprim/sulfamethoxazole.

## Introduction

*Stenotrophomonas maltophilia* is a motile, aerobic, glucose non-fermenting, non-sporulating, Gram-negative bacillus [1], [2]. It can be isolated from various sources in nature, including water, soil, plants, and animals. *S. maltophilia* is primarily an opportunistic human pathogen, causing nosocomial infections in immunocompromised or debilitated patients [3]. It can adhere to moist foreign surfaces and form biofilms. It can thus colonize the inanimate hospital environment and the devices used for patient care [4]. The nosocomial *S. maltophilia* infections are typically polyclonal in origin, except for those acquired in the intensive care unit [ICU] [5]. The main types of infections associated with *S. maltophilia* include pneumonia, bloodstream infections, as well as urinary tract infections, intra-abdominal infections, meningitis, and ocular infections [1]. In patients with cystic fibrosis, *S. maltophilia* can colonize the airways and be associated with acute pulmonary exacerbations [6].

*S. maltophilia* infections are acquiring increasing importance in the hospital environment, as the size of the susceptible patient population increases [7], [8]. The treatment of *S. maltophilia* can be problematic. This pathogen is characterized by intrinsic resistance to multiple classes of antibiotics, owed to various mechanisms such as decreased permeability, production of β-lactamases and of aminoglycoside modifying enzymes, or the presence of multidrug efflux pumps [9]. Resistance can also emerge during therapy [10]. In this context, we sought to study the clinical and microbiological characteristics, as well as the treatment outcome of patients with *S. maltophilia* infection in a general hospital.

## Methods

### Study population

All patients during a 6-year period (Jan 2005–Dec 2010) who provided a culture specimen from which *S. maltophilia* grew were identified retrospectively through the electronic database of the microbiological laboratory of the University Hospital of Heraklion, Crete, Greece. The study hospital is a general, tertiary-care center, with a 680-bed capacity (including an 11-bed ICU). All of the available medical records of the candidate for inclusion patients were retrieved and reviewed. Those patients in whom the *S. maltophilia* isolate was associated with infection were included in the study. Infection versus colonization was differentiated in accordance to the CDC definitions for nosocomial infection surveillance [11]. For patients who had more than one episodes of infection with *S. maltophilia*, only the first episode was analyzed in our study.

### Data extraction

The clinical data extracted for each patient included: demographic characteristics, department of hospitalization, comorbidity, duration of hospitalization, type of infection attributed to *S. maltophilia*, outcome of infection, all-cause in-hospital mortality, and infection-related mortality. The microbiological data extracted included: type of culture specimen, number of positive cultures with *S. maltophilia*, other pathogens isolated in the same as *S. maltophilia* culture specimen or in additional culture specimens during the course of the same episode of infection.

### Definitions

*Malnutrition* was defined as a body mass index <18.5 Kg/m^2^, unintentional weight-loss over 5% of body weight during the last month, or over 10% during the last 6 months. *Trauma* was defined as any kind of injury or infected wound. *Immunosuppression* was defined as the use of immunosuppressive therapy for autoimmune disease, chemotherapy or radiation therapy for neoplasia, use of glucocorticoids, presence of leukemia or lymphoma, HIV-infection, or splenectomy.

### Microbiological testing

Species identification was done using standard microbiological methods (microscopy, culture characteristics, and oxidase reaction), the API-20NE system (bioMérieux, Marcy l'Etoile, France) and the automated Vitek 2 system (bioMérieux). Antimicrobial susceptibility was tested for one *S. maltophilia* isolate per patient with the agar dilution method. The isolates had been kept frozen at −80°C at the microbiological laboratory of the hospital. The agents tested included ceftazidime, ticarcillin/clavulanic acid, amikacin, gentamicin, netilmicin, tobramycin, chloramphenicol, ciprofloxacin, colistin, tetracycline, and trimethoprim/sulfamethoxazole. The results were interpreted according to the Clinical and Laboratory Standards Institute (CLSI) criteria [12]. For agents that specific CLSI criteria for *S. maltophilia* had not been published, the relevant criteria for non-Enterobacteriaceae were used. Specifically for colistin, a minimum inhibitory concentration (MIC) ≤2 mg/L was considered to denote susceptibility, while an MIC ≥4 mg/L was considered to denote resistance. *Escherichia coli* ATCC 25922 and *Pseudomonas aeruginosa* ATCC 27853 were used as quality control strains.

### Treatment

Empirical antimicrobial therapy was defined as the one administered from the beginning of the index infectious episode up to the first isolation of *S. maltophilia*. Targeted antimicrobial therapy was defined as the one administered soon after the first culture results yielding *S. maltophilia* became available. Only the antimicrobial agents that target non-fermenting Gram-negative bacilli with the addition of trimethoprim/sulfamethoxazole were considered of clinical relevance to the treatment of *S. maltophilia* infection and were included in this data analysis. Monotherapy was defined as treatment with only one of these agents. Appropriate antimicrobial therapy was defined as the administration of a regimen that included at least one agent to which the index *S. maltophilia* isolate was susceptible. Resistance to other agents of the same class as well as intrinsic antimicrobial resistance of *S. maltophilia* was taken into consideration for the agents that were not tested [13].

### Patient outcome

The primary outcome for the studied patients was the all-cause, in-hospital mortality. Mortality was considered to be infection-related, when the clinical signs and symptoms of the *S. maltophilia* infection had not resolved at the time of death and the infection directly contributed to death. Cure was defined as the resolution of all symptoms and signs of the infection.

### Statistical analysis

The baseline patient characteristics and treatment parameters were tested for their association with all-cause, in-hospital mortality, using the t-test, the Mann-Whitney U test, or the Fischer's exact test for normally distributed continuous variables, non-parametric continuous variables, and dichotomous variables, respectively. The one-sample Kolmogorov-Smirnov test was used to test the continuous variables for normality. Any variable with a significant association with mortality in the univariate analysis was entered in a multivariate, forward, stepwise (likelihood ratio), logistic regression model. A *p* value≤0.05 was considered to denote statistical significance. All analyses were performed using the SPSS v. 17 software platform (SPPS Inc, Chicago, IL, USA).

## Results

### Baseline patient characteristics

Sixty-eight patients with *S. maltophilia* infection (median age: 70.5 years; 64.7% male) were identified as eligible for inclusion in our study. Four (5.9%) of the 68 included patients developed the index *S. maltophilia* infection while they were outpatients. In all of these 4 patients, the infection was healthcare-associated; 3 had chronic renal failure and the remaining patient had a hematological malignancy. Thirty-six (52.9%) of the 68 included patients were being hospitalized in medical departments (of which 16 in the hematology or oncology department), 18 (26.5%) were being hospitalized in surgical departments, and the remaining 14 (20.6%) were being hospitalized in the ICU, when the episode of *S. maltophilia* infection occurred. All of the included patients had underlying comorbid conditions, except for 3 with *S. maltophilia* keratitis related to the use of contact lenses. The most frequent underlying comorbidity was cardiac disease and malignancy (each observed in 26 [38.2%]), followed by pulmonary disease (in 21 [30.9%]). No patient had underlying cystic fibrosis. A recent or a concurrent infection was noted for 21 patients (30.9%), at the time that the index *S. maltophilia* infection developed. Forty-five (66.2%) of the included patients were immunocompromised, while the majority of those (31 [68.9%]) were receiving treatment with glucocorticoids. Additional data for the baseline characteristics of the studied patients are presented in Table 1.

**Table 1:** Demographic and baseline characteristics of the 68 evaluated patients with *S. maltophilia* infection.

	n (%)
***Demographic characteristics***	
Age (years), [median (IQR)]	70.5 (54–76)
Sex (male)	44 (64.7)
***Department of hospitalization***	
Medical	36 (52.9)
Hematology/oncology	16/36 (44.4)
Surgical	18 (26.5)
ICU	14 (20.6)
***Comorbidity***	
Cardiac disease	26 (38.2)
Malignancy	26 (38.2)
Pulmonary disease	21 (30.9)
Diabetes mellitus	13 (19.1)
CNS disease	9 (13.2)
Renal disease	9 (13.2)
Trauma	9 (13.2)
Musculoskeletal disease	8 (11.8)
Benign prostate hyperplasia	7 (10.3)
Autoimmune disease	6 (8.8)
Hepatic disease	5 (7.4)
Hypothyroidism	3 (4.4)
Prior/concurrent infection with other pathogens	21 (30.9)
***Risk factors for infection***	
Immunosuppression	45 (66.2)
Glucocorticoid treatment	31/45(68.9)
Indwelling catheter, other than CVC*	32 (47.1)
Malnutrition	30 (44.1)
CVC	28 (41.2)
Urinary catheter	26 (38.2)
Surgery	20 (29.4)
Smoking	19 (27.9)
Prior antibiotic therapy (within 1 month)	13 (19.1)
Transfer from another hospital	8 (11.8)
Obesity	5 (7.4)
***Baseline laboratory findings*** [mean (±SD)]	
Hb	13.3 (±1.2)
WBC	11.0 (±1.25)
Neutrophil/WBC percentage	78.9 (±16.2)
***Type of infection attributed to S. maltophilia***	
Respiratory tract infection	37 (54.4)
Bloodstream infection	11 (16.2)
Catheter-related bloodstream infection	4/11 (36.4)
SSTI	7 (10.3)
IAI	6 (8.8)
Peritonitis	3 (4.4)
Cholecystitis	3 (4.4)
Ocular infection	4 (5.9)
UTI	3 (4.4)

Abbreviations: CNS: central nervous system, CVC: central venous catheter, Hb: hemoglobin, IAI: intra-abdominal infection, ICU: intensive care unit, IQR: interquartile range, RTI: respiratory tract infection, SD: standard deviation, SSTI: skin and soft tissue infection, UTI: urinary tract infection.

* Arterial, Swan-Ganz, or nasogastric catheter.

### Types of infection attributed to S. maltophilia

The main type of infection associated with *S. maltophilia* was respiratory tract infection, that was observed in 37 (54.4%) of the included patients; the lower respiratory tract was involved in all of the above patients, except for one who had a deep neck space infection. Eleven (16.2%) patients had a bloodstream infection, in 4 of which it was central venous catheter-related. Seven (10.3%) patients had a skin/soft tissue infection, 6 (8.8%) patients had an intra-abdominal infection (peritonitis and cholecystitis in 3 patients each), 4 (5.9%) had an ocular infection, and the remaining 3 (4.4%) patients had a urinary tract infection.

### Microbiological characteristics

The 68 included patients provided a total of 81 culture specimens from which *S. maltophilia* was isolated. Sixty-one (89.7%) patients provided a single positive culture specimen for *S. maltophilia*, 5 (7.4%) patients provided multiple positive culture specimens of the same type, and the remaining 2 (2.9%) patients provided multiple positive culture specimens of different types. The specific types of the culture specimens that were provided by the patients included in our study and from which *S. maltophilia* grew are presented in Table 2.

**Table 2:** Types of clinical culture specimens from which *S. maltophilia* was isolated.

Culture Specimens	All specimens (N = 74)	Polymicrobial culture specimens (N = 28)
	n (%)	
Bronchial secretions/lavage	23 (31.1%)	5 (17.9%)
Sputum	15 (20.3%)	4 (14.3%)
Pus	8 (10.8%)	4 (14.3%)
Blood	7 (9.5%)	3 (10.7%)
Intravascular catheter tip	4 (5.4%)	1 (3.6%)
Urine	4 (5.4%)	3 (10.7%)
Ascitic fluid	3 (4.1%)	3 (10.7%)
Bile	3 (4.1%)	2 (7.1%)
Contact lense	3 (4.1%)	0
Cornea	1 (1.4%)	0
Peritoneal dialysis fluid	1 (1.4%)	1 (3.6%)
Throat swab	1 (1.4%)	1 (3.6%)
Bone	1 (1.4%)	1 (3.6%)

In 45 (66.2%) of the 68 included patients, the index culture from which *S. maltophilia* infection was diagnosed was monomicrobial. Other Gram-negative pathogens were co-isolated in the index *S. maltophilia–*positive culture in 20 (29.4%) patients, while Gram-positive pathogens were co-isolated with *S. maltophilia* in 14 (20.6%) patients, and *Candida* spp. were co-isolated in 7 (10.3%) patients; detailed relevant data are presented in Table 3. In 24 (35.3%) of the 68 patients, additional pathogens (other than *S. maltophilia*) were isolated in subsequent culture specimens.

**Table 3:** Other microorganisms co-isolated with *S. maltophilia* in the index culture specimen for the diagnosis of *S. maltophilia* infection.

	n (%)
**None**	45 (66.2)
**Gram negative**	20 (29.4)
*Klebsiella pneumoniae*	7 (10.3)
*Pseudomonas aeruginosa*	6 (8.8)
*Acinetobacter* sp.	4 (5.9)
*Achromobacter xylosoxidans*	2 (2.9)
*Escherichia coli*	1 (1.5)
**Gram positive**	14 (20.6)
*Coagulase negative Staphyloccoci*	5 (7.3)
*Enterococcus faecium*	3 (4.4)
*Enterococcus faecalis*	1 (1.5)
*Staphylococcus aureus*	1 (1.5)
*Sterptococcus* sp.	1 (1.5)
*Streptococcus pneumoniae*	1 (1.5)
*Peptosteptococcus pervotii*	1 (1.5)
*Oerskovia sp.*	1 (1.5)
***Candida*** ** spp.**	7 (10.3)

### *In vitro* susceptibility

The antimicrobial agents to which the *S. maltophilia* isolates exhibited the highest susceptibility were colistin (91.2% susceptibility), trimethoprim/sulfamethoxazole and netilmicin (85.3% each), chloramphenicol (83.8%), followed by ciprofloxacin, amikacin, and gentamicin (82.4% each). The susceptibility to ticarcillin/clavulanic acid and ceftazidime was low (26.5% each); detailed relevant data are presented in Table 4.

**Table 4:** Susceptibility pattern of the 68 tested *Stenotrophomonas maltophilia* isolates.

Antimicrobial agents	S (%)	I (%)
Colistin	62 (91.2)	0 (0.0)
Netilmicin	58 (85.3)	4 (5.9)
Trimethoprim/sulfamethoxazole	58 (85.3)	1 (1.5)
Chloramphenicol	57 (83.8)	7 (10.3)
Amikacin	56 (82.4)	3 (4.4)
Ciprofloxacin	56 (82.4)	5 (7.4)
Gentamicin	56 (82.4)	3 (4.4)
Tobramycin	48 (70.6)	1 (1.5)
Tetracycline	47 (69.1)	8 (11.8)
Ceftazidime	18 (26.5)	6 (8.8)
Ticarcillin/clavulanic acid	18 (26.5)	10 (14.7)

I: intermediately susceptible, S: susceptible.

### Antimicrobial treatment

Data on the antimicrobial agents administered for the treatment of the index episode of *S. maltophilia* infection were available for 55 (80.9%) of the 68 included patients. The classes of antimicrobial agents most frequently used as empirical therapy, either as monotherapy or in combination regimens, were the carbapenems (used in 27.3% of the 55 patients), the fluoroquinolones (25.5%), and piperacillin/tazobactam (21.8%). The empirical therapy regimen was deemed inappropriate for 29 (52.7%) patients.

After the first positive culture of *S. maltophilia*, the antimicrobial treatment regimen was modified in 23 (41.8%) of the 55 analyzed patients, including 15 (51.7%) of the 29 patients receiving inappropriate empirical therapy. The classes of antimicrobial agents most frequently used as targeted therapy were the fluoroquinolones (used in 41.8% of the 55 patients), the carbapenems (29.1%), colistin and piperacillin/tazobactam (in 25.5% of the patients each). Trimethoprim/sulfamethoxazole was used in 9.1% of the patients. The fluoroquinolones and the carbapenems were the agents most frequently used as monotherapy (in 9.1% of the patients each). The targeted therapy was deemed inappropriate in 20 (36.4%) of the 55 patients. Detailed data for the antimicrobials used as empirical or targeted therapy in the 55 analyzed patients are presented in Table 5.

**Table 5:** Antimicrobial agents used in the empirical and targeted treatment regimens for 55 patients with *S. maltophilia* infection.

	Use in any regimen	Use as monotherapy
	n (%)	
***Empirical therapy***		
Carbapenems	15 (27.3)	6 (10.9)
Fluoroquinolones	14 (25.5)	3 (5.5)
Piperacillin/tazobactam	12 (21.8)	2 (3.6)
Extended-spectrum cephalosporins*	9 (16.4)	2 (3.6)
Aminoglycosides	9 (16.4)∧	1 (1.8)
Colistin‡	8 (14.5)	0
Tigecycline	5 (9.1)	0
Ticarcillin/clavulanic acid	4 (7.3)	1 (1.8)
Trimethoprim/sulfamethoxazole	3 (5.5)	0
***Targeted therapy***		
Fluoroquinolones	23 (41.8)	5 (9.1)
Carbapenems	16 (29.1)	5 (9.1)
Colistin□	14 (25.5)	0
Piperacillin/tazobactam	14 (25.5)	2 (3.6)
Extended-spectrum cephalosporins*	11 (20.0)	3(5.5)
Aminoglycosides	11 (20.0)∧	1 (1.8)
Tigecycline	7 (12.7)	0
Ticarcillin/clavulanic acid	6 (10.9)	1 (1.8)
Trimethoprim/sulfamethoxazole	5 (9.1)	0

* 3^rd^ or 4^th^ generation cephalosporins.

∧ 4 of the cases refer to ophthalmic use.

□ Data refer to intravenously administered colistin.

### Outcome

Fifty-three (77.9%) of the 68 included patients with *S. maltophilia* infection were cured. Twenty-one (80.8%) of the 26 patients who received appropriate empirical treatment and 23 (79.3%) of the 29 patients who did not receive appropriate empirical treatment were cured. The median duration of hospitalization was 17 days (interquartile range: 9–46 days). Ten (14.7%) of the patients died from any cause during their hospital stay. Death was related to the *S. maltophilia* infection in 3 (4.4%) of the 68 patients. Eight of the 10 total deaths and all 3 of the deaths related to the *S. maltophilia* infection occurred in patients hospitalized in the ICU.

### Risk factors for mortality

In the univariate analyses, the variables that were significantly associated with increased all-cause in-hospital mortality were hospitalization in the ICU (*p*<0.001), presence of a urinary (*p* = 0.005), central venous (*p* = 0.012), or other indwelling catheter (*p* = 0.038), higher white blood cell count (*p* = 0.006), co-isolation of another Gram-negative pathogen in the same culture as *S. maltophilia* (*p* = 0.022), use of tigecycline in the empirical regimen (*p* = 0.011), and use of colistin (*p* = 0.006), tigecycline (*p* = 0.004) and trimethoprim/sulfamethoxazole (*p* = 0.011) as targeted therapy. Hospitalization in the medical ward (*p* = 0.038) and use of fluoroquinolones in the empirical therapy regimen (*p* = 0.041) were associated with decreased mortality. No difference was observed between patients who survived and those who died regarding the administration of appropriate empirical therapy [22 of the 45 (48.9%) patients who survived versus 4 of the 10 (40.0%) patients who died received appropriate empirical therapy, *p* = 0.61] or the administration of appropriate targeted therapy [29 of the 45 (64.4%) patients who survived versus 6 of the 10 (60.0%) patients who died received appropriate targeted therapy, *p* = 0.79). In the multivariate logistic regression model, hospitalization in the ICU was the only variable that was independently associated with mortality (odds ratio: 32.0, 95% confidence interval: 5.3–194.9, *p*<0.001).

## Discussion

In this study, we reviewed 68 cases of *S. maltophilia* infection that occurred in a 6-year period at a tertiary-care, university general hospital, in Crete, Greece. The cases of *S. maltophilia* infection were distributed throughout the hospital departments; the majority occurred in the medical departments (including the hematology and oncology ones). The main type of infection caused by *S. maltophilia* was lower respiratory tract infection, followed by bloodstream infection, skin or soft tissue infection, and intra-abdominal infection. Almost all of the cases had one or more underlying comorbid diseases, the most frequent being cardiac disease, malignancy, and pulmonary disease (although none had cystic fibrosis). The majority of the patients were immunosuppressed, due to either an underlying disease or drug therapy (particularly with glucocorticoids). Many patients also had a recent or concurrent infection with different pathogens or they developed such an infection during their hospital stay.

The *S. maltophilia* isolates collected in our study had relatively high susceptibility to colistin, trimethoprim/sulfamethoxazole, aminoglycosides, fluoroquinolones, good susceptibility to tetracycline, but low to ticarcillin/clavulanic acid and ceftazidime. Still, the empirically administered treatment regimen was microbiologically inappropriate for approximately half of the patients. Even after the culture results became available, the targeted treatment regimen remained inappropriate for approximately one third of the patients. The majority of the patients received combination antimicrobial therapy. The combination regimens most often included agents other than trimethoprim/sulfamethoxazole; the latter was used in less than 10% of the patients. Yet, the all-cause, in-hospital mortality was relatively low (14.7%) and the infection-related mortality was only 4.4%. Hospitalization in the ICU was the only factor that was independently associated with increased mortality.

The spectrum of the clinical syndromes caused by *S. maltophilia* in our study was similar to what has been observed in other relevant studies [14]. Although the incidence of *S. maltophilia* infection may be higher in the ICU, the majority of cases hospital-wide can occur in other departments, due to the higher number of patients in these departments [14], [15], [16]. The affected patients are those with underlying risk factors, such as serious comorbidity, prior use of antibiotics, and prolongation of hospital stay [15]. These are also risk factors for infection with other, more frequently encountered, multidrug resistant Gram-negative pathogens. This was evidenced by the frequent isolation of such pathogens either in the same culture with *S. maltophilia* or in subsequent cultures from the patients included in our study. Many patients received inappropriate empirical treatment against *S. maltophilia*, because piperacillin-tazobactam, extended spectrum cephalosporins or carbapenems, which are commonly used for the empirical treatment of nosocomial infections, do not have substantial activity against *S. maltophilia*.

Still, the targeted therapy was also inappropriate in approximately one third of the included patients. The attending physicians might have chosen to continue administering a regimen to which the patient had responded, even if this was inactive *in vitro*. This could relate to discordance between the *in vitro* activity and the *in vivo* effectiveness of antimicrobial agents or to synergy between the agents administered in combination regimens [10], [16]. Alternatively, the attending physicians might have targeted a presumably more pathogenic organism in the case of a polymicrobial infection. Clinicians without experience with *S. maltophilia* infections might also erroneously assume that this pathogen is susceptible to agents such as the carbapenems, piperacillin/tazobactam or extended-spectrum cephalosporins. This could occur if the antibiogram reports only refer to the relatively few agents to which *S. maltophilia* is not intrinsically resistant and does not specifically state the resistance to the other agents.

Trimethoprim/sulfamethoxazole has been considered as the mainstay of therapy against *S. maltophilia* infections, but this is primarily based on *in vitro* susceptibility data rather than clinical studies [17]. Increasing resistance to trimethoprim/sulfamethoxazole has been reported by several studies [4], [18], mostly related to the horizontal spread of mobile genetic elements carrying resistance genes [19], [20]. In our study, 13.2% of the isolates were resistant to trimethoprim/sulfamethoxazole. Although trimethoprim/sulfamethoxazole was used in only a minority of patients, the mortality associated with the *S. maltophilia* infection was relatively low. This finding suggests that the alternative to trimethoprim/sulfamethoxazole treatment regimens used (mainly combination regimens including a fluoroquinolone, colistin or tigecycline), had good effectiveness.

The fluoroquinolones are one of the main alternatives to trimethoprim/sulfamethoxazole for the treatment of *S. maltophilia* infections [17]. Although the published relevant clinical experience refers primarily to ciprofloxacin, levofloxacin and, particularly, moxifloxacin, can have more potent *in vitro* activity [7], [21], [22], [23]. In our study, the *in vitro* susceptibility to ciprofloxacin was high and a fluoroquinolone was frequently used both as empirical and targeted therapy. Of note, resistance to the fluoroquinolones can arise during therapy [10], [24], [25]. Among the β-lactams, ticarcillin/clavulanic acid and, secondly, ceftazidime are the ones that can be considered as potential therapeutic options against *S. maltophilia* infections, particularly as part of combination regimens [4], [16]. In our study, the susceptibility to both these agents was rather low, in agreement with other relevant studies [26].

The issues presented above highlight the need for new therapeutic options against *S. maltophilia* infections. According to our study, colistin and tigecycline could be considered in this regard. Colistin was the agent to which the *S. maltophilia* isolates had the highest susceptibility, and it was used in a substantial percentage of patients as part of a combination regimen. Variable findings regarding the *in vitro* activity of polymyxins against *S. maltophilia* have been reported in other studies [16], [27], [28]. These differences could relate to differences in the susceptibility testing methods [28]. We used the agar dilution method which is generally considered as the reference standard [29]. Geographical differences in the susceptibility of *S. maltophilia* isolates to polymyxins have also been reported [26], while isolates from patients with cystic fibrosis or patients hospitalized in the ICU can have higher resistance [6]. *S. maltophilia* isolates are frequently susceptible to doxycycline and, particularly, minocycline [30], [31]. The susceptibility to tigecycline (a derivative of minocycline) has also been shown to be high, depending on the interpretative criteria used [26], [30]. Additional clinical experience with the use of tigecycline against *S. maltophilia* infections is currently scarce [32].

The aminoglycosides could also be useful as part of combination regimens for the treatment of *S. maltophilia* infections. [8]. In our study, the susceptibility of the *S. maltophilia* isolates to the aminoglycosides was high, in contrast with most other studies. *S. maltophilia* is considered to have intrinsic resistance to the aminoglycosides that is mediated by various mechanisms, including a chromosomally encoded AAC(6′)-Iz enzyme that inactivates all of the commonly used aminoglycosides, except gentamicin [33]. This enzyme was not present, however, in 33% of 65 isolates in one study, which was associated with substantially low MICs [34].

In our study, both the crude and the infection-related mortality of *S. maltophilia* infection were relatively low. The relevant data in the literature are rather conflicting, but there are studies reporting considerably high attributable mortality of *S. maltophilia* infection [35]. Our study did not include a control group of patients without *S. maltophilia* infection, so we could not formally assess the percentage of deaths attributed to the underlying comorbid conditions. The low mortality observed in our study could relate to the fact that the majority of the patients were hospitalized in other hospital departments than the ICU [36], [37]. Hospitalization in the ICU at the time that *S. maltophilia* infection occurred was the only factor that was independently associated with mortality. This highlights the importance of the severity of the underlying risk factors for the prognosis of patients with *S. maltophilia* infection. We did not analyze indexes of the severity of clinical status in this regard, because these are not commonly recorded in patients not hospitalized in the ICU. Our study also included patients with any type of infection caused by *S. maltophilia*, some of which may be associated with a favorable prognosis.

The main limitation of our study is that the differentiation between colonization and infection with *S. maltophilia* is not always be accurate, particularly in the context of a retrospective study [14], [15]. The pathogenic role of *S. maltophilia* has been debatable, although it is increasingly being recognized as an important pathogen in patients with underlying comorbidity [7]. The pathogenic role of *S. maltophilia* particularly in polymicrobial infections, which constituted a considerable percentage in our study, is also difficult to be ascertained. Other more virulent pathogens may be more important in this regard, and, in any case, polymicrobial infections can have a poorer prognosis [38]. Evaluating the microbiological outcome of *S. maltophilia* infections would be useful to assess the value of different combination regimens, particularly in the case of polymicrobial infections.

In the above context, it is difficult to reliably estimate the clinical effectiveness of the specific antimicrobial agents used for the treatment of *S. maltophilia* infections in our study. Most of the patients received combination antimicrobial therapy. In addition, certain antimicrobial agents might be more or less likely to be used in ICU patients who have a poorer prognosis, which might explain the univariate associations seen between these agents and mortality.

In conclusion, *S. maltophilia* infection can occur in patients with various types of underlying comorbidity and risk factors in the hospital setting. The prognosis of the *S. maltophilia-*associated infection in terms of all-cause mortality can be favorable, except for the patients hospitalized in the ICU. The fluoroquinolones, or potentially colistin and tigecycline, could be useful alternative treatment options to trimethoprim/sulfamethoxazole, mainly as part of combination regimens.
